# Evaluation of teeth injuries in Beagle dogs caused by autoclaved beef bones used as a chewing item to remove dental calculus

**DOI:** 10.1371/journal.pone.0228146

**Published:** 2020-02-13

**Authors:** Caroline Fredrich Dourado Pinto, Willian Lehr, Víviam Nunes Pignone, Caio Peixoto Chain, Luciano Trevizan

**Affiliations:** 1 Department of Animal Science, Universidade Federal do Rio Grande do Sul, Porto Alegre, Rio Grande do Sul, Brazil; 2 AllPet Odonto, Porto Alegre, Rio Grande do Sul, Brazil; 3 Department of Public Administration, Universidade Federal Rural do Rio de Janeiro, Seropédica, Rio de Janeiro, Brazil; University of Illinois, UNITED STATES

## Abstract

Dental calculus (DC) is the most widespread oral problem in domestic dogs. Chewing items are used to remove DC from the tooth surface; they also favor oral health and animal welfare. Raw beef bone mastication also shortly reduces DC in adult dogs. However, it can cause oral lesions and hence is not popular. This study evaluated the impact of bone mastication on the dental roots, enamel, and gingiva of dogs. Twelve adult Beagle dogs were randomly divided into 2 treatment groups in a completely randomized block design: cortical bone (CB) or spongy bone (SB). Intraoral radiographs were obtained on days 0 and 14, and calculus assessment was performed using images captured on days 0, 3, 6, 9, 12, and 14; an integration program was used to measure the proportion between the area covered by calculus and the total area of teeth. DC was completely removed from the first and second premolars and molars from both the arcades in less than 3 days of supplementation, indicating that these teeth were frequently used for chewing (*P* < 0.10). Bones were highly effective for DC removal and gingival inflammation reduction. Despite the hardness of bones, no lesions or teeth root and enamel fracture, or esophageal or intestinal obstructions—complications related to bone ingestion—were noted. However, SB showed some gingival lesions (n = 4) and bone remnants between teeth (n = 2). Gingival lesions were caused by the daily and continuous supply of new pieces of bone for 13 days. Specific pieces of bone should be used for oral home care programs because they shortly remove almost 90% of DC, allowing longer intervals between periodontal cleaning procedures. Long-term studies are required to evaluate the use of bones and evaluate their impact on teeth and periodontium after prolonged supplementation.

## Introduction

The pet food industry has long being focusing on improving the health and longevity of companion animals. Thus, oral health has recently received considerable attention due to the high incidence of periodontal disease. It has been shown that one of the primary contributors to the high incidence of periodontal disease is related to consumption of foods that are less abrasive to the dental surface, thereby facilitating the accumulation of dental plaques and calculus [1, 2, 3, 4]. Periodontal disease directly interferes with the health and life expectancy of companion animals and may affect the function of organs such as heart, kidneys, and liver [5, 6].

Periodontitis, the main oral disease occurring in up to 84% of the canine population of over 3 years of age, is associated with intrinsic factors such as prevalence in dogs of small breeds and progression with age [7]. It is characterized by an inflammatory state, initiated by the deposition of a microbial biofilm—mostly composed of bacteria—on the dental surface, which is known as plaque [8]. Dental plaque extends through the gingival sulcus and, if not removed, undergoes mineralization with calcium carbonates and phosphates present in the saliva, leading to the formation of dental calculus—the main cause of gingivitis. The condition produces irreversible damage in the tissues that provide recoating and support to the teeth, such as the gingiva, alveolar bone, cementum, and periodontal ligament [9]. With progression, mobility and tooth loss occur, causing long-term damage to the dog affected.

Addition of items to the diet, either chemical or abrasive can help modify plaque and calculus deposition [3, 9]. The addition of certain components such as sodium polyphosphates to the diet and snacks is effective in controlling calculus, since it acts as a chelator of salivary calcium, preventing plaque mineralization and deposition on the dental surface [10, 11, 12, 13, 14]; however, it is not likely effective in removing accumulated calculus. The physical properties of the diet, such as abrasiveness, texture, and chewiness, should be considered during food formulation, as they play an important role in plaque control [9]. Several studies have demonstrated that consumption of soft food improved plaque and dental calculus accumulation compared to dry food [3,4].

In addition to food, masticatory items (snacks, bovine skin treats, and bones) can be included in the daily routine of dogs for promoting oral health. Some studies have shown that dogs receiving masticatory items have better oral health, mainly with regard to the removal of dental calculus deposits [15, 16, 17].

Although some dog handlers, trainers and veterinarians had reservations regarding the use of masticatory items as they caused dental fractures, esophageal and intestinal obstructions [18,19,20,21,22,23], Marx et al. [24] showed that the use of specific bones was effective in controlling dental calculus. They evaluated two types of raw bones [cortical bone (CB) vs. spongy bone (SB)] and showed that large accumulations of calculus are removed in the first 3 days of inclusion of masticatory items, with SB removing a higher percentage of calculus in a few days. Moreover, no complications associated with bone consumption were observed. However, the use of raw bovine bones led to concerns about *Salmonella* contamination.

Bone consumption is common in wild animals, especially in wolves, the direct ancestor of domestic dogs [25,26]. Kapoor et al. [27] noted a higher occurrence and severity of calculus and periodontal disease in captive animals that received only boneless ground meat than in free-living animals. These findings emphasize the importance of the mechanical properties of food, which directly contribute to the improvement of oral and systemic health of dogs.

Previous studies evaluated calculus reduction in adult Beagle dogs by supplying raw beef bones, but without diagnosing possible teeth lesions, especially those involving the roots and bone associated with teeth. Because of the concerns about the use of raw bones, this study aimed to compare the dental calculus reduction effect of autoclaved bovine CB from the femur diaphysis with that of autoclaved bovine SB from the femoral epiphysis and analyze their impacts on teeth roots and bone-associated tissues, enamel, and gingiva of adult Beagle dogs.

## Materials and methods

All animal care and handling procedures were approved by the Institutional Animal Care and Use Committee at the Universidade Federal do Rio Grande do Sul (protocol number, 25685).

### Animals and housing

Twelve healthy 4-year-old adult Beagles (6 males and 6 females) from the Animal Science Department, Universidade Federal do Rio Grande do Sul, Porto Alegre, Brazil were used in this study. They were all intact; weighed 12.7 ± 1.67 kg; had a body condition score of 5.3 ± 0.4 out of 9 points [28], measured using a single trained person; and free of endo- and ectoparasites. All dogs were regularly immunized and submitted to clinical and laboratory tests to measure the complete blood count and perform biochemical and coproparasitological analyses before the study was started to ensure that they were healthy. The dogs had never undergone professional dental cleaning and did not receive any regular tooth brushing or food containing entire bones and additives such as sodium polyphosphates to prevent plaques and dental calculus accumulation.

During the study, the dogs were housed in individual stainless steel metabolic cages (1.0 × 1.0 × 1.5 m) equipped with a feces and urine collector, feeders, and drinkers, in a controlled room at 24°C and relative humidity of 70% and a light:dark cycle of 14:10 h.

They were fed twice a day with a non-dental dry extruded complete commercial diet to meet their daily maintenance energy requirements (130 kcal of metabolizable energy × body weight (kg)^0.75^/day), as recommended by the NRC [29]. Water was provided *ad libitum*.

### Treatments

Raw bovine femur was obtained from a commercial slaughterhouse registered and inspected according to Brazilian national laws. The bones were cut using an electric band saw (Implemis IP55; Santa Rosa, Rio Grande do Sul, Brazil) to obtain approximately 4-cm-long pieces of bovine femur from the diaphysis or epiphysis, representing the CB and SB, respectively. Bones were placed in bags before autoclaving (Phoenix Luferco—Araraquara, São Paulo, Brazil) at 1.0 atm for 30 min at 120°C. After autoclaving, the bones were stored at -18°C and thawed at room temperature (20 ± 3°C) before they were offered to the dogs. The bones were offered every morning after the first meal, after the leftovers provided the day before were removed.

### Experimental design

The assay was conducted as a 2 x 5 factorial design consisting of 2 treatments and five intervals, with 3 males and 3 females each, resulting in 6 replicates, which is the minimum recommended by the AAFCO [30]. The treatments included dogs receiving one piece of autoclaved CB or SB per day for 13 consecutive days. The dogs were kept in cages with the bones during 20 h/day and were taken to an outdoor play area for 4 h/day. Before the experiment, the dogs were adapted to the metabolic cages to avoid stress during the assay.

### Sample collection

Oral radiographs of the dogs were obtained on days 0 and 14, and each tooth was evaluated individually. Photographs from the lateral dental arches were obtained on days 0, 3, 6, 9, 12, and 14. One month before the trial was started, all the dogs were adapted to the lateral decubitus position on a table for 3 min per day for capturing photographs. Dogs were held safe by two trained persons and one simulated the photographs; after this assay, the dogs were placed in an outdoor area for socialization. During the trial, the same trained person photographed the dogs’ dental arches, whereas two other people held the dogs in lateral decubitus.

### Calculus assessment

**Fig 1:**
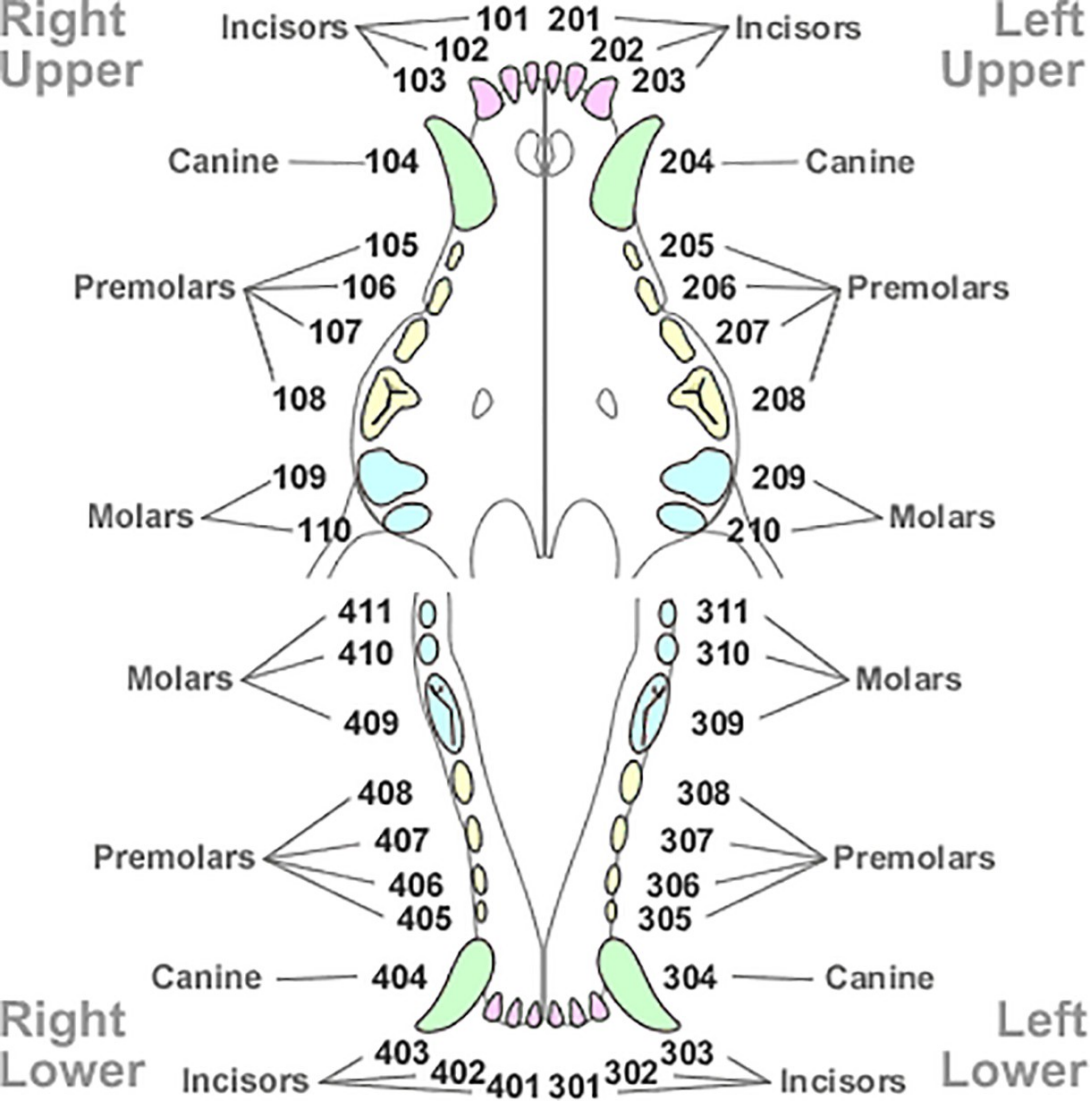
The TRIDAN modified system for dog teeth classification. Adapted from Crossley [32].

The teeth were classified by quadrant in side (left or right), position (maxillary or mandibular), and type of teeth (incisors, canines, premolars, and molars) by using the photographs. Images were obtained using a semi-professional camera fixed approximately 150 mm from the left and right sides of a dog’s dental arches on days 0, 3, 6, 9, 12, and 14. The surface areas of the teeth evaluated were the right maxillary canine (104), left maxillary canine (204), left mandibular canine (304), right mandibular canine (404), right maxillary 1–4 premolars (105, 106, 107, 108), right maxillary 1–4 premolars (205, 206, 207, 208), left mandibular 1–4 premolars (305, 306, 307, 308), right mandibular 1–4 premolars (405, 406, 407, 408), right maxillary molar (109), right maxillary molar (208), left mandibular molars (309, 310), and right mandibular molars (409, 410), according to the teeth nomenclature proposed by the TRIDAN modified system [31] (Fig 1).

Images were analyzed using Image-Pro Plus software for Windows by using the integration surface tool, adapted from Abdalla et al. [33]. Each image was integrated by drawing the outline area of each tooth evaluated, in order to calculate a dog’s total dental arcade area. Next, each image was analyzed by only drawing the outline areas of integrated teeth covered by calculus. The total area was compared with the calculus-covered area by using the same images to determine the proportion of calculus with regard to the total area of teeth, as reported by Marx et al. [24].

### Radiographs

Intraoral radiographs were obtained on days 0 and 14 by a veterinary dentist after the dogs had been anesthetized. The anesthetic protocol administered was 0.03 mg/kg of acepromazine, 2 mg/kg of meperidine, and 4 mg/kg of propofol and isoflurane. The analyzed teeth were then individually radiographed (DIOX602 Portable X-ray System; DigiMed) using an intraoral sensor (Owandy Radiology).

### Statistical analysis

The percentages of dental calculus during the treatment period were analyzed using repeated measures ANOVA for bone type, time, and bone type and time interaction, using Proc Mixed by SAS 9.4 (SAS Inst. Inc., Cary, NC). Means were compared using Tukey’s test at 5% probability (*P* < 0.05). Calculus reduction of each tooth over time was analyzed using panel data regression models, simultaneously considering the 12 observed animals over a time series of 6 periods. The type of bone (CB or SB) and its interaction effects with time were also evaluated in the models. The estimated coefficients and residual normality test were considered significant at 10% probability (*P* < 0.10). Polynomial equations (with quadratic and/or cubic terms) were estimated using Pooled Ordinary Least Squares with Robust Standard Errors by using Gretl 2018c for Windows. Dental evaluations were analyzed by frequency for each treatment. Additionally, the area under the curve (AUC) of each dog was calculated, and the mean of each treatment (CB and SB) was compared by Tukey’s test (*P* < 0.05).

## Results

The dogs showed greater acceptability to the bones. After 20 h, the CB showed minor alterations in the original conformation after being chewed due to their hardness. All CB had teeth marks with complete removal of the bone marrow, revealing their palatability. SBs, having lower density, were reduced to a smaller size than the original, or were completely ingested by the dogs.

The dental calculus was more concentrated in the premolars (105, 106, 107, 108, 205, 206, 207, 208, 305, 306, 307, 308, 405, 406, 407, 408), molars (109, 110, 209, 210, 309, 310, 409, 410), and canines (104, 204, 304, 404). At day 0, the area of calculus with regard to the dental surface of teeth was 56.2% and 62.6% in the dogs of the CB and SB treatment groups, respectively.

Dental calculus was remarkably reduced in all teeth after dogs been in contact with bones (Table 1). The reduction in dogs supplemented with SB was higher from day 3 (57.7% reduction; 19.0% of teeth covered by calculus; *P* < 0.05) until the end of the evaluation, i.e., day 14 (89.5% reduction; 4.52% of teeth covered by calculus; *P* < 0.05), compared to the dogs supplemented with CB on day 3 (35.2% reduction; 29.7% of teeth covered by calculus; *P* < 0.05) until day 14 (64.7% reduction; 10.8% of teeth covered by calculus; *P* < 0.05).

**Table 1:** Dental calculus reduction (%) in adult Beagle dogs receiving two different types of beef bones.

Days	Treatments	
	CB	SB
	Reduction of dental calculus (%)^1^	
0 to 3	35.2±10.8 ^B^^a^	57.7±7.64 ^A^^a^
0 to 6	52.0±8.61 ^B^^b^	72.0±7.90 ^A^^b^
0 to 9	58.9±7.40 ^B^^b^^c^	81.1±5.78 ^A^^cd^
0 to 12	62.7±6.95 ^B^^c^	87.2±4.08 ^A^^cd^
0 to 14	64.7±7.70 ^B^^c^	89.5±4.27 ^A^^d^
Effects	*P*-value	
Treatment	0.0016	
Day	<.0001	
Treatment*day	0.6657	

CB, compact bone; SB, spongy bone.

^1^ Data are expressed as means ± standard deviations.

^a, b^ Means in the same column with different lowercase letters are significantly different (*P* < 0.05).

^A, B^ Means in the same row with different capital letters are significantly different (*P* < 0.05).

**Fig 2:**
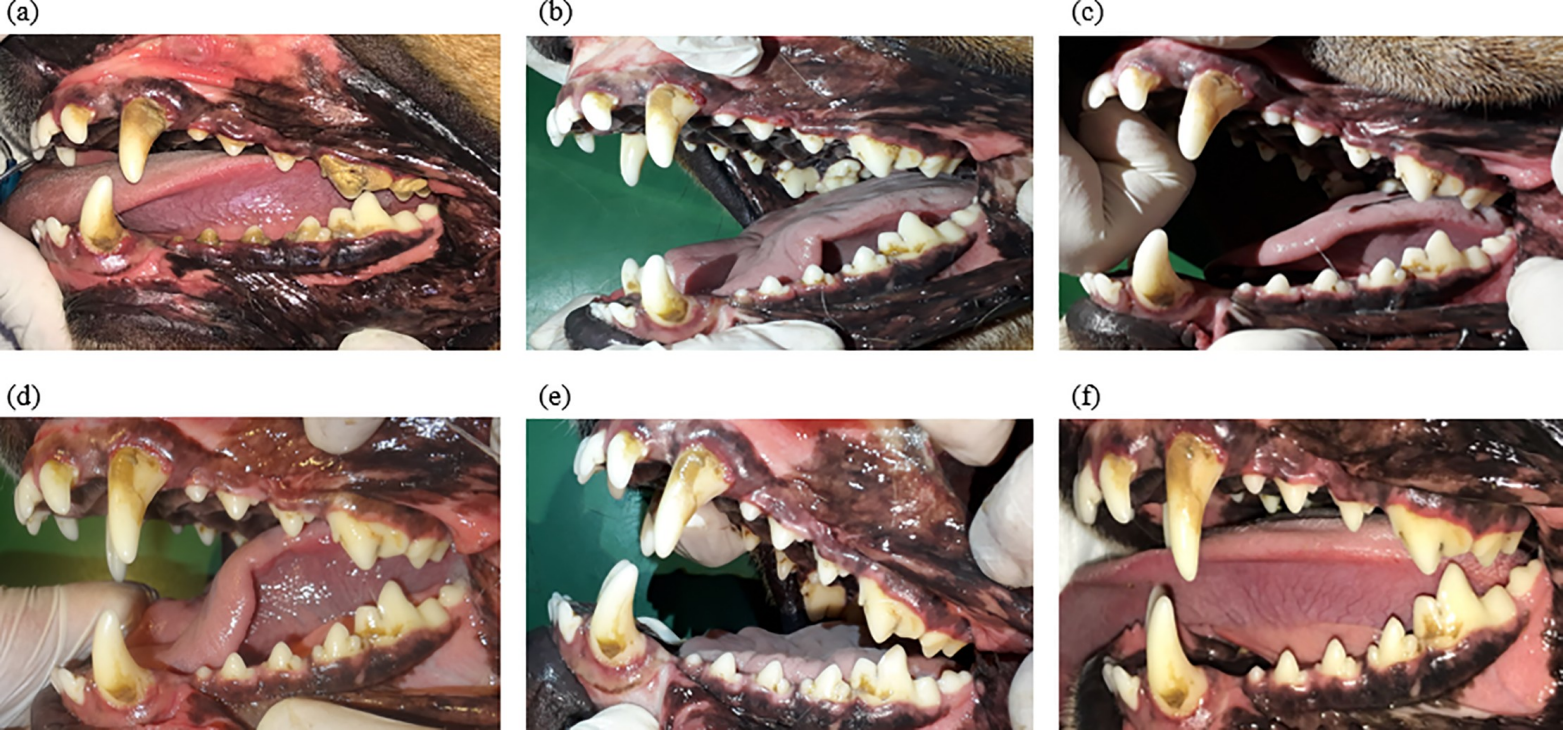
Left dental arcade of the same dog at days 0, 3, 6, 9, 12, and 14 (a–f, respectively) after daily supplementation of autoclaved compact bone.

**Fig 3:**
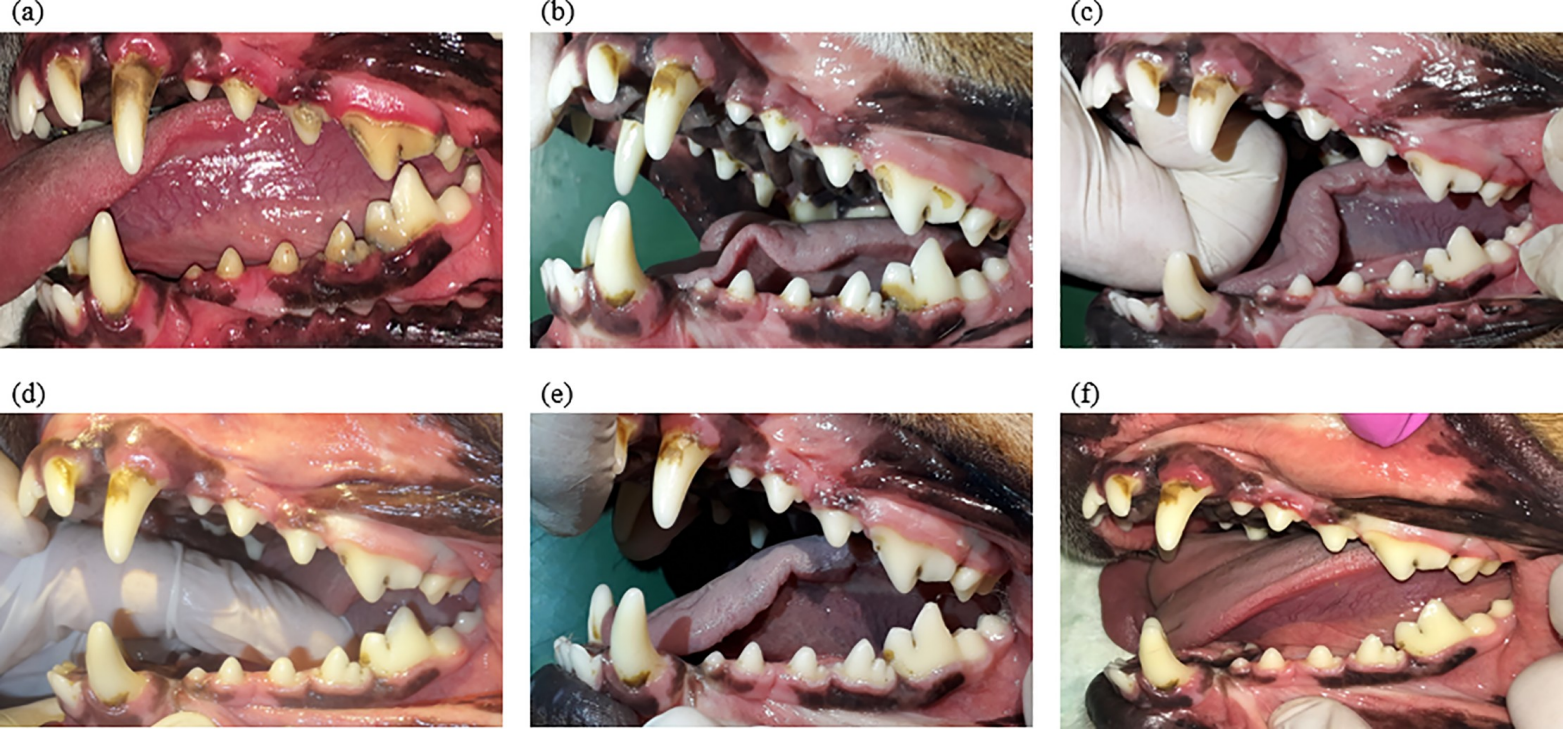
Left dental arcade of the same dog at days 0, 3, 6, 9, 12, and 14 (a–f, respectively) after daily supplementation of autoclaved spongy bone.

In both the groups, dental calculus reduction was noted on day 3 (Figs 2 and 3), highlighting the effectiveness of both the types of bone in the removal of more coarse formations of dental calculus immediately after supplementation was started.

Bone supplementation was effective in the complete removal of all calculus present on teeth 105,106,109, 205, 206, 305, 306, 309, 310, 405, 409, and 410 in less than 3 days after the supplementation had started, making it unnecessary to analyze the calculus reduction over time in these teeth.

**Fig 4:**
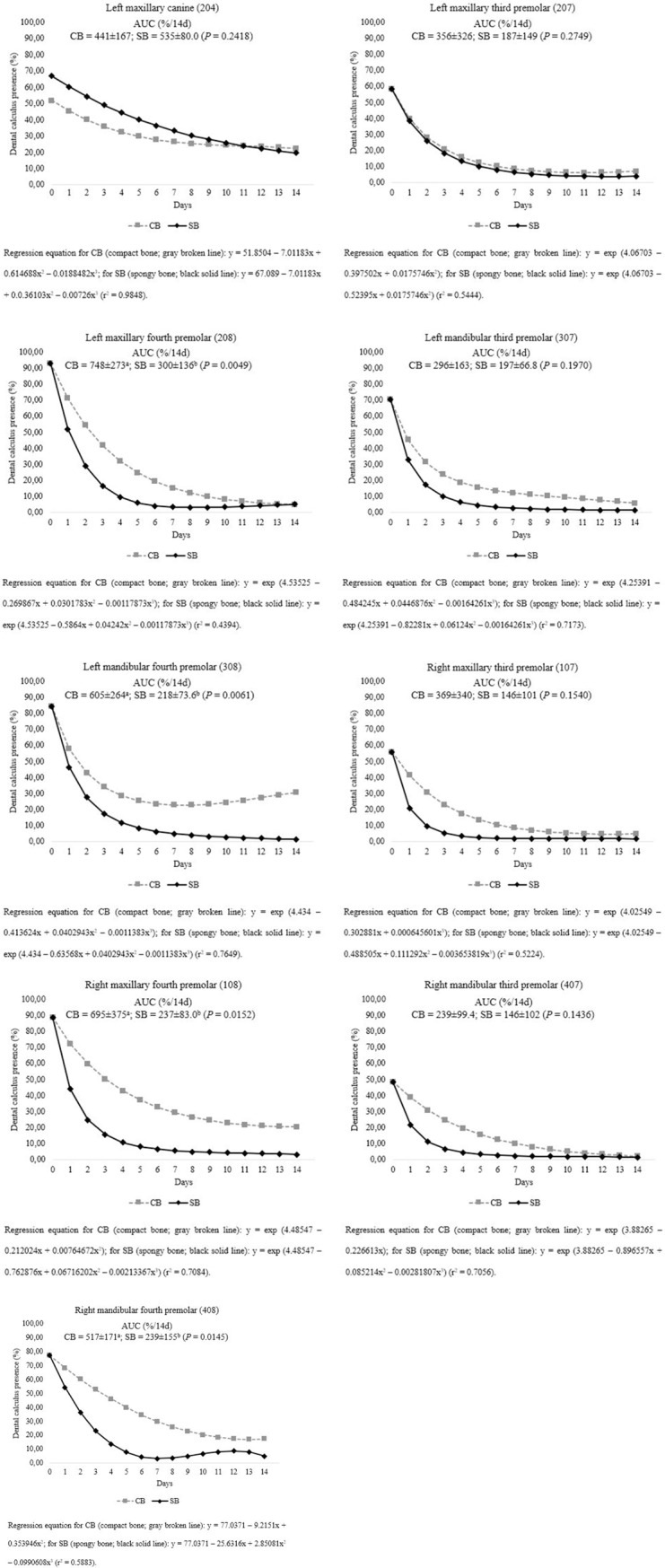
(a) Effect of different types of autoclaved beef bones on dental calculus removal on teeth 204 and 207 during the treatment period in adult Beagle dogs. (b) Effect of different types of autoclaved beef bones on dental calculus removal on teeth 208 and 307 during the treatment period in adult Beagle dogs. (c) Effect of different types of autoclaved beef bones on dental calculus removal on teeth 308 and 107 during the treatment period in adult Beagle dogs. (d) Effect of different types of autoclaved beef bones on dental calculus removal on teeth 108 and 407 during the treatment period in adult Beagle dogs. (e) Effect of different types of autoclaved beef bones on dental calculus removal on tooth 408 during the treatment period in adult Beagle dogs.

The removal of calculus in teeth 107, 108, 204, 207, 208, 307, 308, 407, and 408 varied according to bone type (Fig 4A–4E), with SB having greater effectiveness on cleaning than CB. AUC for teeth 108, 208, 308 and 408 showed differences for type of bone (*P* < 0.05). This effect was prominent in the initial days of supplementation, in which SB was more potent in the removal of calculus, possibly because of its porous characteristic that allowed the penetration of the teeth into the bone matrix, thus facilitating the removal of large deposits of dental calculus. However, with time, calculus removal also became prominent in the group supplemented with CB, indicating that even hard bones have the ability to remove dental calculus.

**Fig 5:**
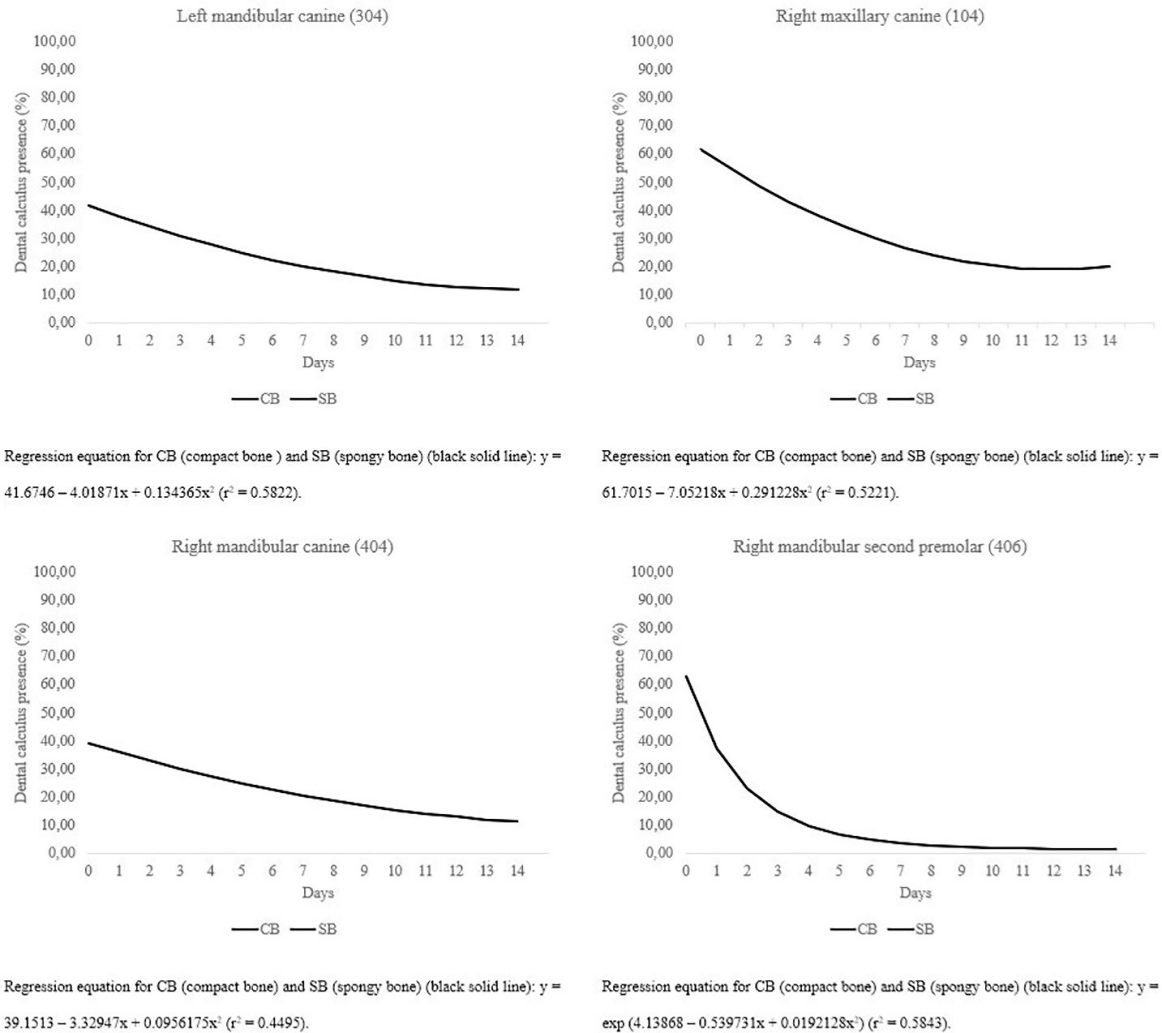
(a) Effect of time on dental calculus removal on teeth 304 and 104 during the treatment period in adult Beagle dogs receiving different types of autoclaved beef bones. (b) Effect of time on dental calculus removal on teeth 404 and 406 during the treatment period in adult Beagle dogs receiving different types of autoclaved beef bones.

Dental calculus reduction on teeth 104, 304, 404 and 406 was similar regardless of the density of bones provided to the dogs (*P* > 0.10; Fig 5A and 5B), and time was the predominant factor in the removal of dental calculus. For these teeth, especially canines, the structure of bone matrix provided it is not important, once these teeth are preferentially used to seize foods.

**Fig 6:**
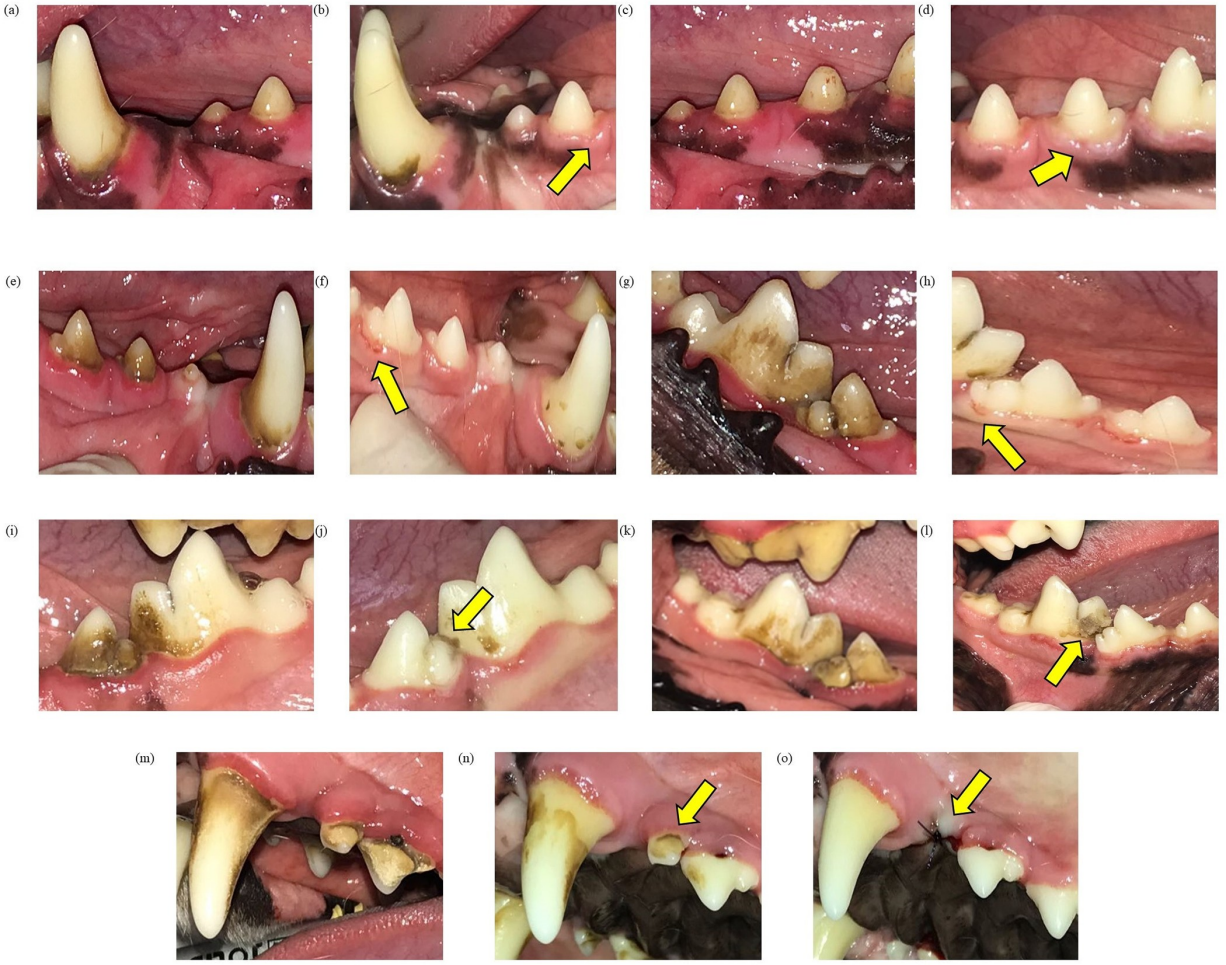
Macroscopic oral lesions in adult dogs supplemented with SB. (a) Day 0. (b) Day 14, gingival traumatic injury on 306. (c) Day 0. (d) Day 14, gingival traumatic injury on 307. (e) Day 0. (f) Day 14, gingival traumatic injury on 407. (g) Day 0. (h) Day 14, gingival traumatic injury on 408. (i) Day 0. (j) Day 14, presence of bone between teeth 308 and 309. (k) Day 0. (l) Day 14, presence of bone between teeth 408 and 409. (m) Day 0. (n) Day 14. (o) Day 14, after prophylaxis tooth extraction on 205.

Macroscopic evaluations indicated the occurrence of some lesions only in the SB treatment group (n = 7; Table 2). Gingival traumatic lesions were prevalent. Other lesions observed included the presence of pieces of bones between teeth in two dogs and one dental extraction on day 14 of treatment (this dog had extensive alveolar bone loss and dental mobility degree II on day 0) (Fig 6). Dogs use premolars to chew bones; hence, a preexistent lesion on these teeth probably contributed to the final extraction.

**Table 2:** Number and localization of dental injuries in adult Beagle dogs after supplementation of bones with different densities for 13 days.

	Lesions	Treatments	
		CB	SB
Gingiva	Traumatic lesions	-	306, 307, 407, 408 (n = 4)
Others	Presence of bone between teeth	-	308–309, 408–409 (n = 2)
	Tooth extraction	-	205 (n = 1)
	Total	-	7

CB, compact bone; SB, spongy bone.

The teeth most affected by chewing of bones were the premolars and molars. No endodontic lesions were observed, such as dental dimming, periapical abscess, and pulp chamber or root canal enlargement. As expected, no dog showed enamel or root fractures due to the continuous supplementation of bones. Moreover, complications such as esophageal and intestinal obstructions were not observed in the dogs during the experimental period.

## Discussion

This study aimed to evaluate the effect of supplementation with specific autoclaved bones of different hardness on dental calculus removal, and how this would impact the oral and dental health of adult dogs, based on the ability of the bones to promote the removal of large deposits of dental calculus by causing friction on the dental surface. Bovine raw compact and spongy bones are effective in removing large deposits of dental calculus over a short time, improving oral health and wellbeing of adult Beagle dogs, as reported by Marx et al. [24]; however, the injuries that may occur on the gingiva, enamel, and roots of teeth have not yet been evaluated.

In this study, we expected that supplementation with autoclaved CB and SB would promote intense dental calculus removal, similar to that observed with raw CB and SB, while minimizing the risk of *Salmonella* transmission because of the sterilization of the bones before they were offered to the dogs. We hypothesized that supplying autoclaved bones would be beneficial in reducing dental calculus without causing lesions on the enamel, roots, and gingiva of adult Beagle dogs. Radiographs were performed on all dogs on the day before the bone supplementation was started, which revealed only one lesion in one dog. This dog had an extensive alveolar bone loss and dental mobility on tooth 205 prior to bone supplementation. Radiographs were performed again 14 days after the experiment had started; hence, the lesions reported were exclusively from 14 days of chewing bones.

Both the types of bones used in this study were highly effective in removing dental calculus. Differences according to the type of bone were significantly distinct since the beginning of the supplementation, with prominent dental calculus reduction of 57.7% in the SB group between days 0 to 3 (62.6% to 19.0% of teeth covered by calculus) compared to 35.2% presented by the CB group between the same days (56.2% to 29.7% of teeth covered by calculus). The difference between the type of bones remained until the last day of evaluation, with the SB group showing 89.5% of dental calculus reduction between days 12 to 14 (5.45% to 4.52% of teeth covered by calculus) and the CB group showing 64.7% of reduction between the same interval (11.9% to 10.8% of teeth covered by calculus).

As reported by Marx et al. [24], the difference in the dental surface cleaning potential between the bones can be attributed to the distinct histological characteristics of the epiphysis and diaphysis of the femoral bone. The SB allows the teeth to penetrate in the matrix, thereby increasing the surface contact between the tooth and bone and promoting sufficient friction to remove thick deposits of calculus in a few days. Conversely, the CB, owing to its hardness, requires more time and mechanical work to promote similar cleaning.

The masticatory behavior of dogs interfered with the dental calculus removal. Dogs preferred to use premolar and molar teeth to chew than the canine teeth. This is because of the different functions assigned to the teeth according to their morphology. Premolar and molar teeth are responsible for mastication and grinding because they have a broad and flat surface, whereas canines serve to seize and tear the food towing to their conical shape [34].

Besides the characteristics of teeth, another factor that positively influences the oral health of domestic dogs is the physical properties of food, especially texture. The physical properties of food, especially texture, positively influence the oral health of domestic dogs. Dry foods promote abrasion to the dental surface because of the need of mechanical forces such as more intense apprehension and chewing, unlike soft foods, which do not promote abrasion, thereby facilitating dental plaque accumulation and calculus formation [9]. Gawor et al. [3] found that the incidence of lymphadenopathy (81.8% vs. 54.8%), dental calculus (44.3% vs. 17.2%), and periodontitis (77.8% vs. 45.3%) was higher in dogs fed moist diets than in those fed a dry diet. Buckley et al. [4] observed that the oral health index of dogs fed moist and home diets deteriorated, increasing the probability of developing oral diseases. However, dogs in this study had consumed dry food since they were puppies, and hence, the percentage of dental calculus was high at day 0 of the experimental period, revealing the low effectiveness of kibbles in promoting friction. Although dry foods do not promote the complete removal of dental plaques and calculus, or effectively prevent periodontitis, their physical characteristics are beneficial in promoting abrasiveness during mastication compared to soft foods, directly affecting the improvement of oral health.

The use of dietary supplements and treats that promote oral health caused substantial reduction in dental plaques and calculus. Polyphosphates are known to prevent dental plaque and calculus formation as they are chelators of calcium salts present in the saliva, avoiding the mineralization of plaques, and thus reducing the incidence of dental calculus [35]. Previous studies have revealed that coating biscuits with 0.6% hexametaphosphate decreased dental calculus formation by 46–80% over a 4 week period [10,11]. Carciofi et al. [12] demonstrated that biscuits coated with 0.6% sodium pyrophosphate reduced dental calculus index by 18.9% after 4 weeks of supplementation. Pinto et al. [14] found that dental calculus was reduced by 24.2% when kibbles were coated with sodium tripolyphosphate, and it was reduced by 34.2% and 47.6% when sodium hexametaphosphate was added to mash and applied as coating to kibbles, respectively. Not only has it been shown that dental diet significantly decreases dental calculus, but the use of masticatory items has also been shown to be an effective oral hygiene tool as it helps in the removal of dental plaque and calculus. Quest [36] found that plaque, calculus, and halitosis were reduced in dogs provided one dental chew daily for 28 days. Stookey [17] reported that the use of a soft rawhide chew item for 4 weeks in Beagle dogs reduced calculus by 28.2%; plaques, by 18.5%; and gingivitis, by 45.7%, compared to that in the control group.

As mentioned above, numerous alternatives are available for oral health maintenance and prevention; however, no method is completely efficient in the total removal of plaques and dental calculus. Periodontal treatment, executed by a specialized professional, allows the complete cleaning of the teeth and must be executed periodically. A regular home oral care program should be established as a preventive measure, thereby prolonging the interval between teeth cleaning procedures. Daily tooth brushing is the most effective home-based method for the removal and control of dental plaques. A study evaluating the effect of the frequency of brushing teeth showed that daily brushing or alternate–day brushing was beneficial in controlling plaque and calculus accumulation in Beagle dogs, reducing the severity of pre-existing gingivitis [37]. However, teeth need to be brushed using a specific type of toothbrush, ensuring that the gingival tissue is not damaged [38]. In addition, daily brushing demands time and commitment from trainers, and hence reduces adherence to the procedure.

Among the several alternatives mentioned above to control dental plaques and calculus, the superior results obtained in the present study show the high potential of bone supplementation in the oral health maintenance of adult dogs. Veterinarians and trainers restrict the adoption of bones in the diet, because several studies have shown the association of the consumption of bones with chocking, visceral perforation, and esophageal and intestinal concretion formation [19,20,21,22,23]. However, consumption of carcasses containing bones is a common habit in wild dogs and wolves, and the occurrence of dental calculus and oral diseases is extremely low in those species [26]. In addition, bone supplementation improves animal welfare owing to the time spent during chewing [39]. Colyer [40] analyzed 1157 wild canid skulls and demonstrated that only 2% of the sample had periodontal disease suggested by alveolar bone destruction. Though, a recent study reported an increase in tooth fracture in gray wolves, the direct ancestor of domestic dogs, when the prey:predator ratio decreases due to increased bone consumption and difficulty in capturing prey [41].

Accidents with bones in dogs are related with the type and size of bones consumed. This trial was conducted using specific bones that were cut into pieces and were considered to be safe for Beagle dogs to avoid swallowing, offered in the morning and recovered after 20 h. Moreover, each day a new piece of bone was offered. This allowed a high level of control, which could be a limitation of this study, as it may be difficult to replicate this at home with dogs under different care.

Dental injuries in dogs usually result from fights with other animals, falls, vehicular accidents, and chewing of hard objects such as stones and bones [42]. The lateralized chewing behavior shown by dogs increased the incidence of lesions on the premolar and molar teeth, especially of the upper arch. No fractures on the roots and enamel of teeth were observed during the evaluation of arches by using photographs or radiographs, this may be due to the short period of bone supplementation that was not enough to assess the possible impact on these structures. The main lesions (n = 4) from bone supplementation were noted in the gingival tissue. These could be attributed to the constant abrasion applied by the bones to the gingival tissue.

Excessive friction promotes the migration of the gingival margin in the apical direction, primarily forming gingival fissures or clefts that can heal without permanent damage. However, the maintenance of mechanical forces on the marginal gingival tissue leads to the formation of recessions or more severe gingival retractions. In humans, improper tooth brushing with force and the use of toothbrushes with hard bristles increases the incidence of gingival fissures [43]. In this study, the incidence of gingival injuries was attributed to the continuous friction between the bone and the tissue. However, these injuries could also result from the masticatory action for other chewing items or even toys. Despite the gingival traumas caused by the impact of bone chewing, continuous bone supplementation for 13 days reduced the pre-existing redness and gingival edema noted on day 0. Thus, mechanical removal of dental calculus improved the health of gingival tissue.

Although there was an improvement in the visual appearance of the gum, there was no reduction in plaque and calculus under the gumline. The maintenance of subgingival plaque and calculus is the etiological factor of loss of dental adhesion to its alveolus, characteristic to periodontal disease. Thus, bones are not efficient in removing plaque and calculus under the gumline, they are only able to remove it on the crown.

The consistency and porosity of SB allowed greater mechanical action, facilitating breakage, which justifies the presence of bone between the teeth in two dogs in our study. The permanence of these pieces may produce a foreign body reaction in a long-term, causing damage to the health of these animals.

Supplementation with bones of different textures revealed contrasting results regarding the removal of dental calculus, with greater efficiency of SB but not enough to remove plaque and calculus under the gumline. Despite the prevalence of gingival lesions, no fracture or bone resorption was observed at the teeth roots. Bones can be used as a regular home oral care program, considering their high effectiveness in removing thick deposits of dental calculus within a few days after starting supplementation. CBs were not associated with lesions or dental fractures, as expected. They cleaned efficiently, but required more time to reach the same calculus reduction level as that promoted by SB. Although CB may take longer to remove dental calculus compared to SB, they may be the most suitable type of bone for home oral care program, as breaking the bone into small pieces is difficult. Bone chewing does not promote the removal of plaque or dental calculus from the subgingival surface, but its efficiency in removing supragingival plaque and calculus allowed the prolonging of intervals between periodontal cleaning. Long-term studies are warranted to evaluate the impact of CB on dental structures.

## Conclusions

Supplementation of bones, especially SB, was highly effective in removing supragingival calculus in adult Beagle dogs. As expected, continuous consumption of bones for 13 days did not cause any root fracture, enamel fracture, and bone resorption. Bones promote reduction of almost 90% of dental calculus over the teeth and need to be associated with regular oral prophylaxis. Pieces of SB may be stocked between teeth and need to be removed to avoid injuries. The long-term safety of bones as chewing items needs to be evaluated by conducting further studies, in order to determine the impact of continuous and long-term mastication on the teeth and periodontium. Moreover, complementary studies are required to develop chewing items with porosity close to SB in order to increase the variety of efficient masticatory items to improve the cleanliness and maintenance of canine oral health.
